# WeChat app-based reinforced education improves the quality of opioid titration treatment of cancer-related pain in outpatients: a randomized control study

**DOI:** 10.1186/s12885-020-07270-w

**Published:** 2020-09-04

**Authors:** Zhiyou Peng, Lin Li, Yuan Chen, Zhiying Feng, Xiangming Fang

**Affiliations:** 1 Department of Pain Medicine, the First Affiliated Hospital, Zhejiang University School of Medicine, Hangzhou, China; 2 Department of Anesthesiology, Yuyao people’s Hospital, Ningbo, China; 3 Department of Anesthesiology, Hangzhou first people’s Hospital, Hangzhou, China; 4 Department of Anesthesiology, the First Affiliated Hospital, Zhejiang University School of Medicine, Hangzhou, China

**Keywords:** WeChat app, Opioid titration treatment, Cancer-related pain, Outpatients

## Abstract

**Background:**

As inadequate pain communication contributes to difficulties in optimizing outcomes of outpatients, we investigated the effect of reinforced education using WeChat App to the opioid titration treatment of cancer-related pain in the outpatient setting.

**Methods:**

We conducted a prospective study to compare reinforced education using Wechat with care as usual from February to December 2019. Patients in the reinforced education group received reinforced education via Wechat, while those in the control group received care as usual. Effect measurements for both groups are carried out with questionnaires at the baseline and 3 days later. Questionnaires include pain intensity (NRS), treatment-related adverse events, cancer-related quality of life (QOL), sleep (PSQI), satisfaction, anxiety (GAD-7) and depression (PHQ-9). Number of patients whose NRS reduced to less than three points in 24 h was the primary outcomes. Secondary outcomes included treatment-related adverse events, cancer-related quality of life, sleep, satisfaction, anxiety and depression.

**Results:**

Although there was no significant difference regarding pain intensity (NRS) between the two groups at 72 h, the rate of NRS that reduced to less than three points in 24 h was significantly higher in the Wechat group than in the control group. Patients’ satisfaction was significantly higher in the Wechat group than in the control group. There was no significant difference between the two groups regarding the other findings at 72 h, including pain intensity (NRS), cancer-related quality of life (QOL), anxiety (GAD-7), depression (PHQ-9), and sleep (PSQI). However, no significant difference was found between the two groups for constipation, nausea, vomiting, dizziness, somnolence, pruritus, loss of consciousness, and death.

**Conclusions:**

Our results indicated that receiving instructions delivered by Wechat resulted an increased number of patients with good pain control and better satisfaction. The study provided insight into the effectiveness of the reinforced education using a Wechat app delivered by a doctor to outpatients in the titration treatment of cancer-related pain.

**Trial registration:**

This study was registered at chictr.org (Registration number: ChiCTR1900021150, Date of Registration: January 30, 2019).

## Background

Pain is the most significant concerns of many patients secondary to their disease and significantly impacting upon their quality of life. It is a considerable challenge to management of this pain successfully, especially for outpatients. Following the World Health Organization (WHO) analgesic ladder, treatment with strong opioids can be considered the mainstay of cancer pain therapy [1, 2]. However, many terminal cancer patients prefer to be at home than anywhere else. There is an increasing requirement of family carers’ role in managing cancer-related pain and providing palliative care at home [3]. However, cancer patients at home are less likely to have access to adequate analgesia compared to those in hospital [4, 5]. As pain is a subjective experience, patients who experience pain at home cannot get timely treatment if they cannot recognize and deal with it correctly or cannot contact the doctor in time.

Adequate knowledge and understanding of pain and analgesic medications is critical for managing cancer pain, and influences on the quality of pain management for patients at home [6]. Distinguishing and handling risk factors for adverse reactions, the titration of the dose based on pain level and knowing where to obtain additional information are the foundation of effective cancer pain management. Considering these challenges and the barriers that have been identified in the outpatient setting, patients need to be supported with reinforced education through more comprehensive and timely help [7, 8]. Thomas ML, et al. promoted education or motivational-interviewing-based coaching compared to usual care to improve cancer pain management [9]. Improving titration treatment of cancer-related pain through traditional communication routines, including face-to-face verbal education, written booklets or visual aids or telephone-based re-instruction, has proven useful [10]. However, the effectiveness and applicability of these interventions have been shown to be inconsistent. So, newly healthcare technology is promising in facilitating telemonitoring to enhance education management.

In recent years, Wechat provided an easily accessible and interactive channel of communication between patients and medical providers, playing an important role in optimizing clinical work [11, 12]. As a pain monitor has been identified as an important barrier to adequate pain management in the outpatient setting, we tested the hypothesis that reinforced education with Wechat is promising in terms of enhancing the cancer pain management of outpatients.

## Methods

A prospective, randomized, controlled study was conducted at the First Affiliated Hospital, Zhejiang University School of Medicine. The study was approved by the institutional review board of the first affiliated hospital, Zhejiang University School of Medicine, and was registered at chictr.org (ChiCTR1900021150). Written informed consent was obtained from all patients and our study adhered to CONSORT guidelines. According to the WHO’s three-step principle, individuals over the age of 18 years of age and not older than 75 years who needs to start opiate titration in outpatient clinics were enrolled in the study, which took place from February to December 2019.

Patients meeting the following inclusion criteria were eligible to participate: subjects voluntarily signed the informed consent for the clinical observation; Subject had a digital score of persistent pain (≥4) and (or) number of breakthrough cancer pain (≥3 times per day) originated from cancer, regardless of distant metastasis; pain is mainly caused by physical factors, not mental and psychological factors; subjects or caregivers can use Wechat skillfully. We defined breakthrough cancer pain as “a transient exacerbation of pain that occurs either spontaneously, or in relation to a specific predictable or unpredictable trigger, despite relatively stable and adequately controlled background pain” [13]. Patients were excluded if they met the following exclusion criteria: had a history of alcohol abuse, drug or opium abuse; new anti-cancer drugs or new radiotherapy schemes were used during clinical observation; cognitive deficits or mental disorders, or consciousness disorders; pregnancy or lactation; morphine-related contraindications; patients with unstable vital signs; patients participating in another interventional clinical study.

According to the order of the patients enrolled, the patients were assigned to the Wechat group or the control group according to the computer-generated random numbers plan.

### Protocol of opioid titration treatment

**Fig. 1:**
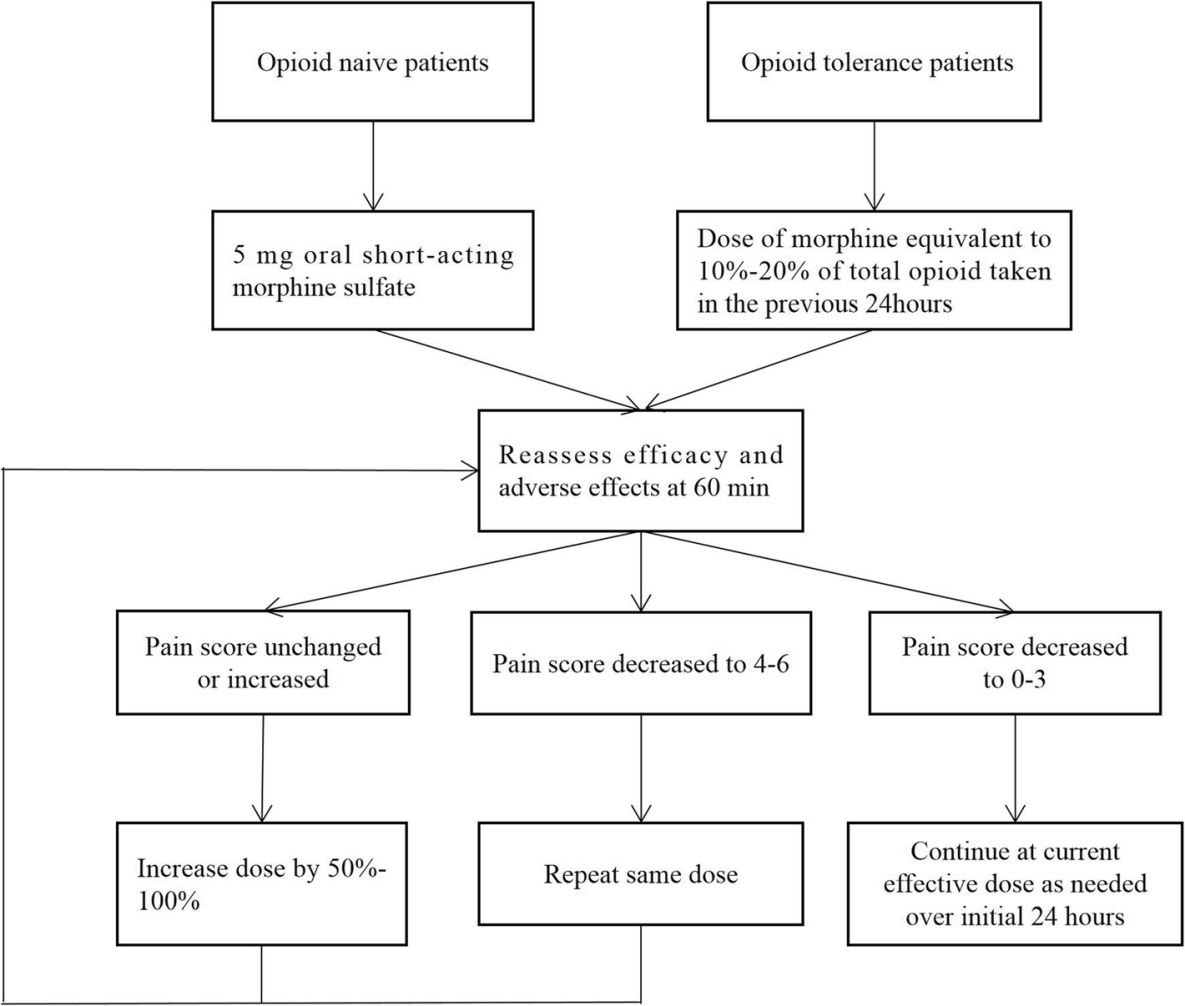
The schedule of opioid titration treatment

The National Comprehensive Cancer Network’s (NCCN) clinical practice guidelines in adult cancer pain, this protocol defines the dose of oral morphine, the interval, absence of limitation of the total dose, numerical rating scale (NRS) threshold required to administer morphine, criteria to stop titration, and how to convert immediate-release morphine into sustained-release oxycodone. Figure 1 describes the titration schedule. Morphine titration is administered until a NRS ≤ 3 is reached, or until the onset of a serious adverse events. Patients themselves recorded the pain score and adverse events in their record book. Patients were told to go to nearby medical institutions to seek help if they experienced adverse events. Patients considered opioid tolerant were those who had been taking for a week or longer at least 60 mg of morphine daily, or at least 30 mg of oral oxycodone daily, or an equianalgesic dose of another opioid. Pain scores was evaluated every 4 h and morphine immediate-release tablets were given when the NRS score was greater than 3. The sustained dose was half of the total amount of morphine immediate-release tablets and oxycodone sustained-release tablets in the first 24 h. The rescue dose is 10–20% of the total dose of drugs in the first 24 h [14, 15].

### Education

Any questions were answered at the initial appointment. Reinforced education was provided by one experienced doctor through Wechat. Based on the standardized management of cancer pain in the control group, Wechat was used to provide reinforced education. Wechat Subscription Platform was used to publish information including the pain score, knowledge related to opioid analgesics, role of such drugs, common errors when taking tablets, possible adverse reactions, and prevention and treatment methods. Doctors actively and timeously answered patients’ concerns regarding the treatment, and marked the department contact telephone on the subscription plat information column. Members of the pain control group used Wechat to communicate using either voice or text depending on the patient’s needs in the micro-letter. We tried to enable all participants to achieve satisfactory pain control and handle risk factors related to opioid use.

Other than answering questions at the initial appointment, patients in the control group received no further active education from doctors. Afterward, patients were seen again at the outpatient clinic for further education or contacted by phone for a follow-up consultation. The timing and frequency of the follow-up consultation differed according to patients’ general conditions.

Patients in the reinforced education group received reinforced education via Wechat, while those in the control group received care as usual. Wechat Subscription Plat is used to publish information, which includes pain score and opioid analgesics related knowledge. Doctors actively answered patients’ doubts in the treatment in time. We strived to let all participants be able to achieve satisfactory pain control and how to handle risk factors related to opioid using. The intervention period and the control period before study assessments were conducted was 72 h the initial appointment.

### Outcome assessments

Number of patients whose NRS reduced to less than three points in 24 h was the primary outcomes. Secondary outcomes included pain intensity (NRS), satisfaction with pain management and pain intensity, sleep (Pittsburgh sleep quality index, PSQI), anxiety (Generalized anxiety disorder-7, GAD-7), depression (Patient health questionnaire-9, PHQ-9), and cancer-related quality of life (QOL) at 72 h after the initionation of treatment. In addition, treatment-related adverse events including nausea, vomiting, dizziness, somnolence, pruritus, loss of consciousness and death were also recorded.

#### GAD-7 and PHQ-9

We used the GAD-7 and PHQ-9 to measure the anxiety and depression. The reliability and validity of GAD-7 and PHQ-9 are well established and they are commonly used as self-report tools to evaluate the severity of anxiety and depression. Patients whose scores are greater than 4 on the GAD-7 or the PHQ-9 are considered as suffering from anxiety or depression, respectively [16, 17].

#### PSQI

The PSQI was used to evaluate patients’ sleep quality of patients. This scale consists of 18 self-report items that are divided into 7 dimensions, namely subjective sleep quality, time to fall asleep, sleep duration, sleep efficiency, sleep disturbances, use of sleep medication, and daytime dysfunction. The score on each dimension ranges from 0 to 3 and higher total scores indicate lower sleep quality [18].

#### QOL

The QOL was used for evaluating the quality of life of cancer patients. This scale consists of 12 self-report items, including appetite, spirit, sleep, fatigue, pain, family coordination, colleagues coordination, oneself understanding of cancer, attitude to treatment, daily life, side effects of treatment, facial expressions. The score on each dimension ranges from 1 to 5 with the total scores as 60 and higher total scores indicate better quality of life.

### Statistical analysis

Patients in the Wechat and control groups were expected to show a 15% improvement (from 75 to 90%) in the rate of NRS ≤ 3 within 24 h. Thus, a sample size of 130 patients in each group was required for a statistical power of 80% at a two-tailed significance level of 0.05. Assuming a 10% dropout rate, it was deemed that 145 patients per group would be required to obtain statistically significant results for the primary outcome.

Continuous variables were presented as means ± standard deviation, and categorical data were shown as numbers and percentages. For different variable types, we employ different approaches to evaluate their differences. For categorical variables, we employ Chi-squared tests to evaluate the variable differences. While for continuous variables, we adopt Student’s T test to assess their differences. The statistical significance is measured by *p* value, and *p* < 0.05 indicated statistical significance. All analyses were performed using the SPSS software (Ver. 24.0; IBM Corp., New York, USA).

## Result

**Fig. 2:**
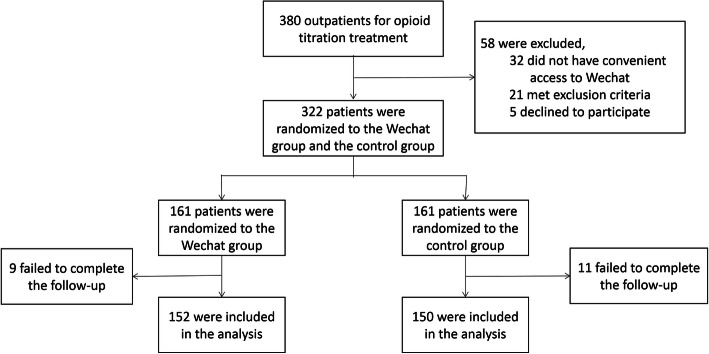
Patients recruitment flowchart

Between February and December 2019, 380 outpatients were considered for the purposes of this study. Figure 2 illustrated the patient recruitment flowchart. The baseline characteristics of two groups were similar, including the age, gender, level of education, pain site, and message receiver (refer to Table 1).

**Table 1:** Baseline characteristics of the study patients

		Wechat group (*n* = 152)	Control group (*n* = 150)
Age (years), mean (SD)		55.6 ± 6.8	56.3 ± 7.0
Female (n)		80 (52.6%)	78 (52.0%)
Smoke history (n)		27 (17.8%)	24 (16.0%)
Hypertension (n)		33 (21.7%)	28 (18.7%)
Diabetes (n)		12 (7.9%)	14 (9.3%)
Level of education (n)	Elementary school or less	20 (13.2%)	22 (14.7%)
	High school	92 (60.5%)	90 (60.0%)
	College or higher	40 (26.3%)	38 (25.3%)
Pain site (n)	Abdomen	92 (60.5%)	93 (62.0%)
	Chest	38 (25%)	36 (24.0%)
	Limb	12 (7.9%)	14 (9.3%)
	Head	10 (6.6%)	7 (4.7%)
Message receiver	Patients	32 (21.1%)	33 (22.0%)
	Caregivers	120 (78.9%)	117 (78.0%)

Although there was no significant difference regarding pain intensity (NRS) between the two groups at 72 h, the rate of NRS that reduced to less than three points in 24 h was significantly higher in the Wechat group than in the control group. Patients’ satisfaction was significantly higher in the Wechat group than in the control group. There was no significant difference between the two groups regarding the other findings at 72 h, including cancer-related quality of life (QOL), anxiety (GAD-7), depression (PHQ-9), and sleep (PSQI) (all *p* > 0.05) (refer to Table 2).

**Table 2:** Effect of reinforced education with Wechat on the outcome of cancer pain management at 72 h. T0: baseline, T1:72 h after treatment, *p < 0.05

Outcome measures	Wechat group (n = 152)		Group Control (n = 150)	
	T0	T1	T0	T1
Pain intensity (NRS)	6.8 ± 1.9	2.5 ± 1.2	6.6 ± 2.1	2.8 ± 1.1
Cancer-related quality of life (QOL)	35.2 ± 6.5	46.3 ± 5.5	36.3 ± 6.2	43.4 ± 5.2
Anxiety (GAD-7)	16.1 ± 3.3	9.8 ± 2.2	16.3 ± 3.6	12.4 ± 3.1
Depression (PHQ-9)	17.2 ± 3.0	10.1 ± 2.8	16.7 ± 3.4	11.2 ± 2.2
Sleep (PSQI)	17.2 ± 4.2	12.7 ± 3.3	18.4 ± 4.6	14.6 ± 3.9
NRS that reduced to less than 3 (n)		146 (96.1%)		122(81.3%)*
Satisfied patients (n)		130 (85.5%)		112(74.7%)*

*NRS* numerical rating scale; *QOL* cancer-related quality of life; *GAD-7* Generalized anxiety disorder-7; *PHQ-9* Patient health questionnaire-9; *PSQI* Pittsburgh sleep quality index. Continuous variables were presented as means ± SD, and categorical data were shown as numbers and percentages

However, no significant difference was found between the two groups for constipation, nausea, vomiting, dizziness, somnolence, pruritus, loss of consciousness, and death (refer to Table 3).

**Table 3:** Treatment-related adverse events reported during the procedure. *p < 0.05

Outcome measures	Wechat group (n = 152)	Control group (n = 150)
Constipation	20 (13.2%)	22 (14.5%)
Nausea	10 (6.6%)	12 (8.0%)
Vomiting	7 (4.6%)	9 (6.0%)
Dizziness	5 (3.3%)	8 (5.3%)
Somnolence	0	1 (0.7%)
Pruritus	1 (0.7%)	0
Loss of consciousness	0	0
Death	0	0

## Discussions

The study provides insight into the effectiveness of education reinforced with Wechat delivered by a doctor to outpatients on the titration treatment of cancer-related pain. Our results indicated that patients receiving instructions delivered by Wechat had a relatively higher quality of opioid titration treatment and better satisfaction.

As actual cancer pain management in outpatient settings have different kinds of problems in practice, clinicians urgently need of a promising solution for follow-ups with outpatients to strengthen pain medication management [19, 20]. Although information on the use of opioids for improving the opioid titration treatment of cancer-related pain can be easily found on the internet, it’s not easy for patients to get what they really need. After evaluated a technology-based multicomponent self-management support intervention that combines the monitoring of pain, adverse effects, and medication with graphical feedback, education, and nurse support, it was found that self-management support results in better pain control and better quality of life than care as usual [21]. The results of a systematic review of intervention studies suggests that educational interventions delivered face-to-face, supported by written and/or other resources, and by appropriate follow-ups can potentially improve family carers’ knowledge and self-efficacy for pain management and reduce attitudinal barriers [8]. To the bet of our knowledge, this is the first study demonstrating that Wechat, as an adjunct to regular instructions, improves the opioid titration treatment of cancer-related pain in outpatients. This may be because Wechat allowed patients with more access to knowledge related to treatment, ensured that patients knew how to seek help, and ensured that they received a timely response when suffering severe pain or adverse drug reactions.

Our results indicated no significant difference regarding pain intensity (NRS) between the two groups at 72 h; however, the rate of NRS that reduced to less than 3 points in 24 h was significantly higher in the Wechat group than in the control group. Patients’ satisfaction with pain management and pain intensity was significantly higher in the Wechat group than in the control group. There were no significant differences between the two groups regarding the other findings at 72 h, including cancer related quality of life (QOL), anxiety (GAD-7), depression (PHQ-9) and sleep (PSQI).

The frequent occurrence of unwanted side effects may be a barrier to optimal dosing and patients’ compliance, especially for patients at home. It has been suggested that the goal of the opioid titration treatment of cancer-related pain in outpatients is to find a favorable balance between pain control and side effects [22, 23]. However, no significant scores were found for two groups for constipation, nausea, vomiting, dizziness, somnolence, pruritus, loss of consciousness, and death. The majority of the adverse effects were mild to moderate in severity, with similar profiles for the two groups. This may be mainly because our research mainly focuses on the incidence of adverse events, while the treatment of these adverse reactions needs a certain period of time. In the future, we need to pay attention to the effect of wechat education on the treatment of adverse reactions. Taken together, these results suggest the beneficial effect of reinforced education using Wechat in the management of the opioid titration treatment of cancer-related pain in outpatients.

Compared to care as usual, reinforced education using Wechat result in better pain control and better patients’ satisfaction, contributing to improve cancer pain in outpatients. The intervention could be used for other cancer-related health problems or pain problems in other chronic disease populations [8, 21, 24]. Patients require assistance to easily access information on pain and pain medication, and on when and how to get help [25, 26]. Patients also need to be able to recognize and monitor pain and adverse events to gain insight into their own situation and feedback about how they are doing. In the current period of virus epidemic, Wechat may also optimize the pain management and prevents cross-infections among medical workers and patients, which may help to alleviate shortages in the medical workforce to fight COVID-19 on the front line [27].

This study has some limitations. As this study mainly evaluated the effect of Wechat-based reinforced education on opioid titration, the follow-up time was only 3 days. In the future, evaluating the long-term help of Wechat for cancer pain management should be considered. In addition, although we try to obtain patient’s information through data acquisition and analysis in order to obtain better clinical protocols, it is impossible to do big data analysis due to the limitation of the number of patients. However, this may be a good research direction in the future. Better programs should be developed to apply to a larger number of people, so as to do big data analysis and artificial intelligence analysis to obtain the optimal diagnosis and treatment automatically in the future, which will better serve for public health.

## Conclusions

Our results indicated that patients receiving instructions delivered by Wechat had a relatively higher quality of opioid titration treatment and better satisfaction. To provide long-term support for a wider population of cancer patients with pain, clinicians should integrate evidence-based activities to support reinforced education in routine clinical practice.

## Data Availability

The data used to support the findings of this study are available from the corresponding author upon request.

## Abbreviations

NRS: Numerical rating scale
QOL: Cancer-related quality of life
GAD-7: Generalized anxiety disorder-7
PHQ-9: Patient health questionnaire-9
PSQI: Pittsburgh sleep quality index
WHO: World Health Organization
NCCN: National Comprehensive Cancer Network
